# Iopromide CT peritoneography for diagnosis and management of dialysate scrotal leakage in continuous ambulatory peritoneal dialysis

**DOI:** 10.1186/s12882-026-04901-5

**Published:** 2026-03-27

**Authors:** Zhibin Xie, Huyan Yu, Jian Lin, Qing Ye

**Affiliations:** Department of Nephrology, Zhongshan City People’s Hospital, Zhongshan, 528403 China

**Keywords:** Peritoneal Dialysis (PD), Mechanical complications of peritoneal dialysis, Peritoneal dialysate leak, Scrotal leak, Computed tomography peritoneography (CTp), Clinical case report

## Abstract

**Background:**

Scrotal leakage of dialysate, a serious complication in Continuous Ambulatory Peritoneal Dialysis (CAPD) patients, results from elevated intra-abdominal pressure. It increases infection risk, impairs ultrafiltration, and reduces quality of life. Conventional diagnostic methods often lack sensitivity and dynamic assessment, leading to delayed management.

**Objective:**

To evaluate the efficacy of iopromide CT peritoneography (CTp) in diagnosing scrotal dialysate leakage and its role in guiding clinical decisions, and to summarize the management pathway.

**Methods:**

Eight CAPD patients with suspected scrotal leakage between August 2020 and August 2025 underwent CTp. Clinical baseline characteristics, dialysis duration, symptom onset, and diagnostic-therapeutic timelines were analyzed.

**Results:**

CT peritoneography accurately identified the sites and tracts of dialysate leakage, facilitating prompt clinical intervention. A multidisciplinary management strategy, incorporating individualized dialysis prescription adjustments and subsequent laparoscopic repair, was associated with successful leak resolution and favorable patient outcomes in this case series. Commonly observed clinical features in our case series included overweight, hypertension, and hypoalbuminemia.

**Clinical trial number:**

(Project No.: 2025-100)

**Supplementary Information:**

The online version contains supplementary material available at 10.1186/s12882-026-04901-5.

## Introduction

Continuous Ambulatory Peritoneal Dialysis (CAPD) is a widely used treatment option for patients with end-stage renal disease. First introduced in 1976, this technique remains commonly employed in modern clinical practice [1, 2]. CAPD can lead to various complications, including those of infectious and non-infectious origin. Scrotal leakage of peritoneal dialysate is a serious complication, primarily caused by increased intra-abdominal pressure due to dialysate infusion [1]. It can result in multiple adverse outcomes: the accumulation of glucose-rich fluid in the scrotum significantly raises the risk of infections such as scrotal abscess and peritonitis[ [3]; impaired ultrafiltration may necessitate a reduction in dialysis dose, transition to intermittent peritoneal dialysis, or even temporary or long-term hemodialysis, thereby increasing the healthcare burden [4]. Additionally, symptoms including scrotal swelling, pain, walking difficulties, and skin irritation severely compromise the patient’s quality of life.

However, early and accurate diagnosis of leakage remains challenging. Conventional methods have significant limitations: physical examination has low sensitivity for minor leaks and difficulty distinguishing leakage from hernia or hydrocele [5]; conventional CT lacks dynamic imaging capability and often fails to clearly depict leakage pathways (e.g., patent processus vaginalis); ultrasound is operator-dependent and insensitive to low-flow leaks; although radionuclide imaging (e.g., with ⁹⁹ᵐTc-labeled albumin) can detect leakage, it offers poor spatial resolution, unable to precisely locate the leak site and anatomical route, while also exposing patients to radiation [6]. These methods share common shortcomings including insufficient sensitivity, ambiguous localization, and inability to dynamically visualize leakage tracts, often leading to delayed diagnosis and management of occult leaks, which represents a critical hidden risk for CAPD treatment failure. Accurate and rapid localization of the leakage site is crucial for successful treatment, yet current techniques often fall short.

Computed tomography peritoneography (CTp) has been commonly used to evaluate peritoneal dialysis-related complications [1, 7]. It was first introduced in 1979 to visualize the peritoneal cavity and study intra-abdominal fluid dynamics [8]. Compared to ultrasound, X-ray, conventional CT, MRI, MR peritoneography, and scintigraphy, CTp offers several advantages in diagnosing PD complications, such as avoiding interference from intestinal gas, detecting abnormalities missed by conventional CT or ultrasound, short scanning time, high contrast resolution, detailed anatomical visualization, wide availability, and relatively low risk [9–12]. Although some publications have highlighted the importance and efficacy of this method, there remains a scarcity of systematic and comprehensive studies sharing full procedural experience and summarizing the entire management pathway for CAPD-associated scrotal leakage.

Therefore, we conducted a retrospective analysis of 8 CAPD patients clinically suspected of scrotal dialysate leakage. Data on clinical baseline characteristics, time since initiation of peritoneal dialysis, onset of scrotal leakage, and the complete diagnostic and treatment timeline were collected. This study aims to investigate the clinical features of these patients, evaluate the application value of iopromide CT peritoneography in diagnosis and management, and summarize experiential insights based on the current clinical pathway for managing scrotal leakage.

## Methods

### Study population

A retrospective analysis was conducted on 8 CAPD patients clinically suspected of peritoneal dialysate scrotal leakage between August 2020 and August 2025 in the Department of Nephrology, Zhongshan City People’s Hospital. All patients underwent CT peritoneography using iopromide (370 mg iodine/mL) mixed with dialysate. Data including clinical baseline characteristics, time of peritoneal dialysis initiation, onset of scrotal leakage, and the complete diagnostic and treatment timeline were collected.

### Imaging protocol

**Fig. 1:**
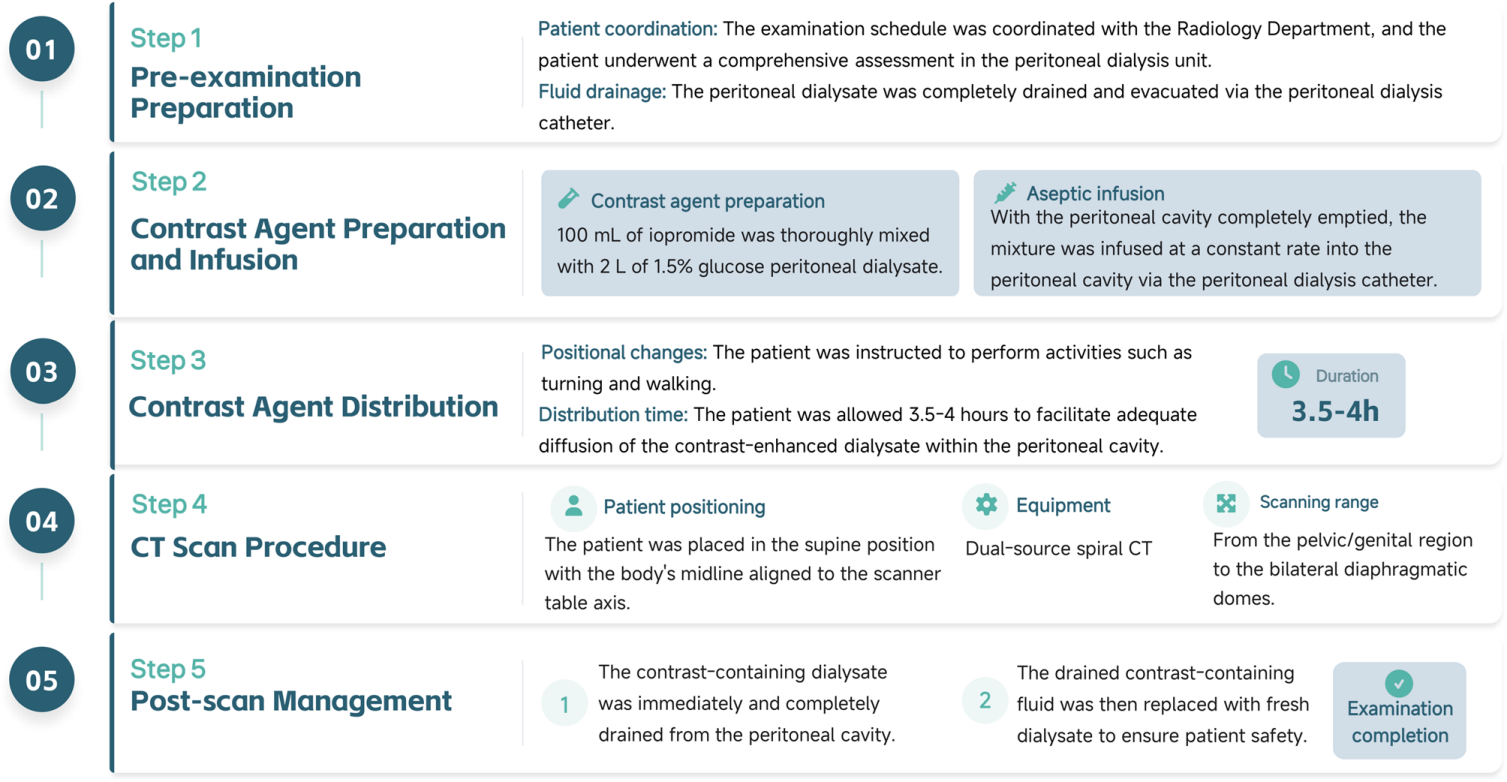
Imaging Protocol of CT Peritoneography

The Standardized CT Peritoneography Procedure comprises five parts, as illustrated in Fig. 1.

**Fig. 2:**
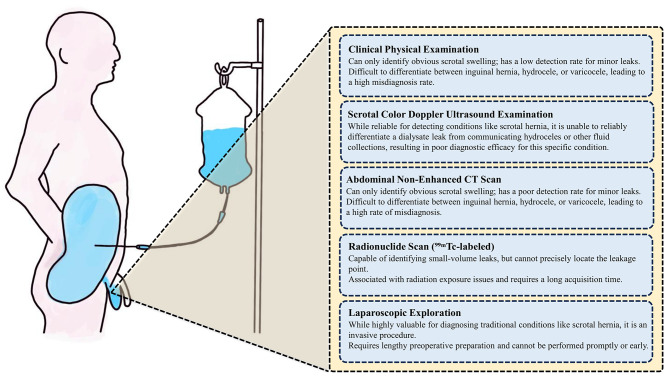
Comparative analysis of different diagnostic modalities for scrotal leakage

**Fig. 3:**
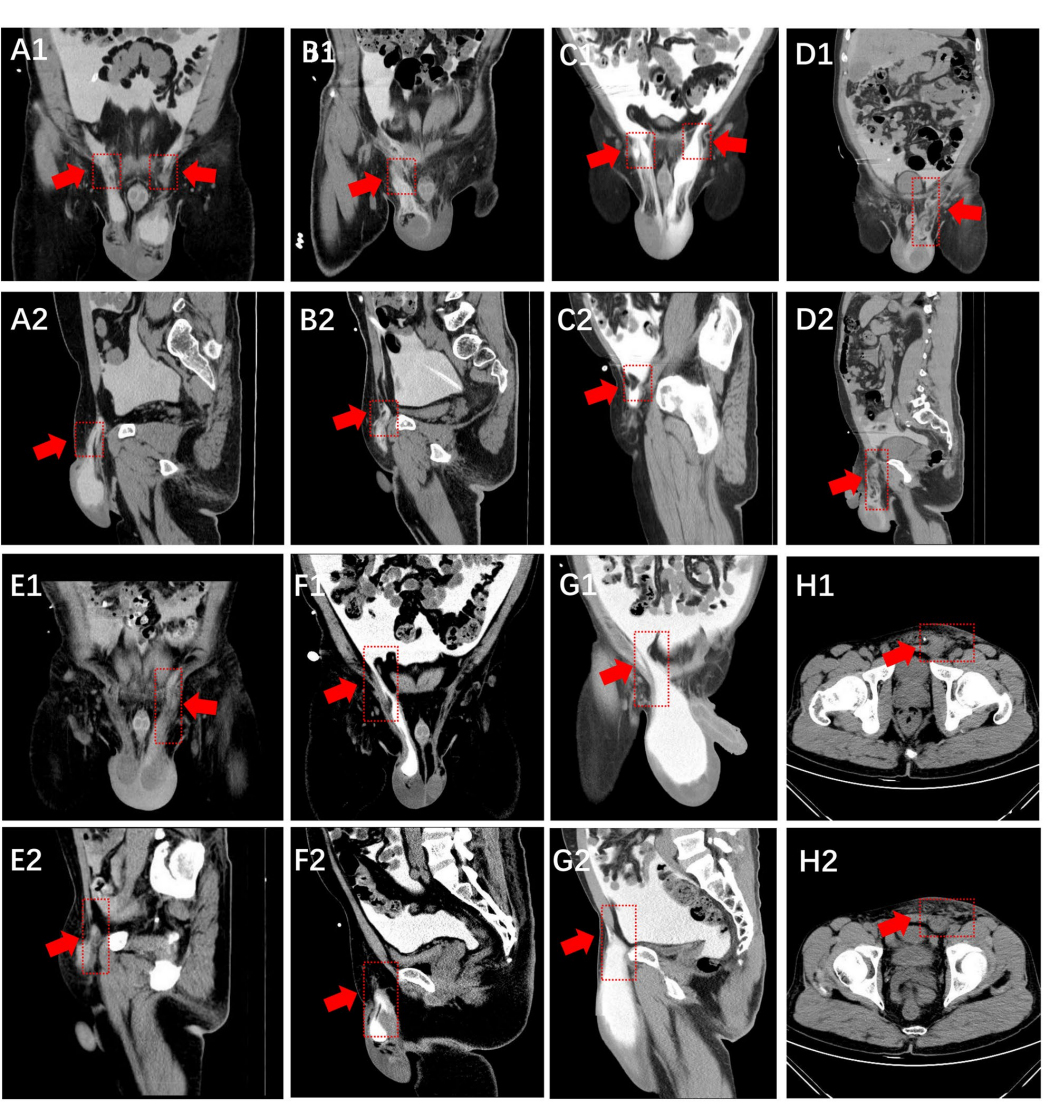
Summary of Imaging Findings from CT Peritoneography for Diagnosis and Leakage Pathways in 8 Cases of Scrotal Leak. Label 1 indicates imaging results in the coronal plane. Label 2 indicates imaging results in the sagittal plane. (Labels H1 and H2 are unique, representing imaging results in the axial plane for Case 8. This was our first patient undergoing CT peritoneography. The image quality is inferior to the subsequent seven cases, likely because our early inexperience led to initiating the CT scan immediately 30 min after contrast agent injection. For the later 7 patients, the contrast agent was allowed to remain in the peritoneal cavity for 2–3 h, which significantly improved imaging quality.). Labels A through H correspond to Case 1 through Case 8, respectively. For detailed radiological descriptions and all corresponding images, please refer to Supplement Appendix 1

**Fig. 4:**
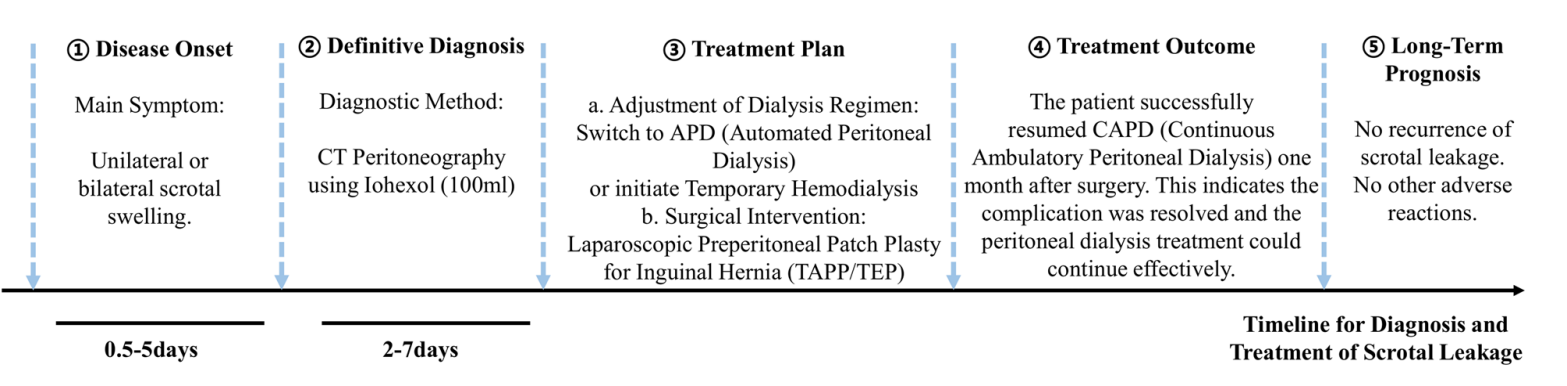
Clinical management timeline for scrotal leak in peritoneal dialysis patients

#### Pre-examination preparation

Following coordination with the Radiology Department, the patient was evaluated in the PD unit. Prior to the examination, the peritoneal dialysate was completely drained via the PD catheter.

#### Contrast preparation and infusion

Under strict aseptic conditions, 100 mL of a non-ionic contrast agent (iopromide, 370 mg I/mL) was thoroughly mixed with 2 L of 1.5% glucose peritoneal dialysate. With the peritoneal cavity completely emptied, the mixture was infused at a constant rate into the peritoneal cavity via the PD catheter by a specialized nurse in a sterile treatment room. The infusion volume was similar to or slightly greater than the patient’s conventional dialysis dose.

#### Contrast distribution

After infusion, the patient was instructed to perform positional changes (e.g., turning, walking) for 3.5 to 4 h to promote adequate diffusion and distribution of the contrast-enhanced dialysate within the peritoneal cavity.

#### CT scanning

The patient was placed in the supine position with the body axis aligned to the scanner midline. Imaging was performed using a dual-source spiral CT scanner, with the scan range covering the pelvic genital area to both diaphragmatic domes.

#### Post-scan management

Upon completion of the examination, the patient returned to the ward. The contrast-containing dialysate was immediately drained completely and replaced with fresh dialysate.

### Data analysis and image interpretation criteria

#### Data analysis

Relevant clinical data were collected, including baseline characteristics, peritoneal dialysis initiation time, scrotal leakage onset, and full treatment timeline. A baseline table was constructed, and disease-related features along with the diagnostic-therapeutic process were summarized.

#### Image interpretation criteria

The diagnostic criteria were established with reference to previously published standards [1, 7]. The key criterion for diagnosing scrotal leakage on CT peritoneography is the direct visualization of contrast-enhanced dialysate passing through a patent processus vaginalis into the inguinal canal and subsequently into the scrotum. The imaging findings include: (1) Direct signs: This is the definitive feature for diagnosis. CT images clearly demonstrate the abnormal pathway of contrast-enhanced dialysate along the following trajectory (Origin: from the peritoneal cavity; Route: through the deep inguinal ring into the inguinal canal, descending along its course; Endpoint: accumulation within the scrotum). (2) Discriminatory value: The presence of contrast within the pathway (processus vaginalis/inguinal canal–scrotum) confirms scrotal edema resulting from dialysate leakage, allowing differentiation from other causes (e.g. inflammation or infection).

### Treatment planning and follow-up

Post-diagnosis treatment strategies and follow-up outcomes were compiled. Data on the occurrence of related adverse events and clinical benefits were summarized and analyzed.

## Results

### Diagnostic advantages of CT peritoneography and comparative analysis with existing diagnostic methods

The differential diagnosis of scrotal swelling in patients undergoing peritoneal dialysis encompasses several possibilities, including: dialysate scrotal leakage, infectious lesions (such as scrotal abscess, epididymo-orchitis), non-infectious hydrocele, inguinal hernia (with or without content), hematoma, neoplastic lesions, and lymphatic reflux disorders. Among these, dialysate scrotal leakage, being a complication specific to PD, requires accurate identification for critical therapeutic decision-making.

Compared to traditional diagnostic methods, CT peritoneography plays an indispensable role in ruling out or confirming dialysate leakage. While ultrasonography can detect scrotal fluid accumulation, its sensitivity is limited for low-flow leaks, and it inadequately visualizes the complete anatomical pathway of leakage. Conventional non-contrast CT may indicate the presence of fluid but cannot dynamically demonstrate communication between the fluid and intraperitoneal dialysate. Furthermore, techniques like radionuclide imaging suffer from insufficient spatial resolution.

Through systematic comparison of various diagnostic methods reported in previous literature (Fig. 2), this study demonstrated that CT peritoneography can directly and dynamically visualize the complete pathway of contrast-enhanced dialysate from the peritoneal cavity through the processus vaginalis and inguinal canal into the scrotum. This high-resolution anatomical delineation capability is of paramount importance in the differential diagnosis of scrotal swelling. The method not only confirms dialysate scrotal leakage by demonstrating characteristic contrast migration pathways but also effectively rules out the diagnosis by excluding abnormal contrast distribution, thereby providing clear direction for clinical diagnosis and treatment.

### Baseline characteristics of enrolled patients

A total of 8 patients were included in the analysis. Data on age, BMI, and medical history (such as diabetes and hypertension) were extracted. The baseline characteristics of the patients are detailed in Table 1. The mean age was 45.38 ± 10.39 years, and the mean BMI was 25.64 ± 2.47 kg/m². Some patients had pre-existing conditions including diabetes and hypertension.

**Table 1:** The characteristics of participants in this study

Case	Gender	Age (year)	BMI (kg/m^2^)	Hypertension (yes/no)	DM (yes/no)	Surgery history (yes/no)	Constipation (yes/no)	Hb (g/L)	ALB (g/L)	Ca (mmol/L)	*P* (mmol/L)	PTH (pg/mL)	K (mmol/L)	Cr (umol/L) admission
1	Male	36	22.2	Yes	No	Yes	No	113	37.7	2.06	1.37	316	3.03	1626
2	Male	46	22.8	Yes	Yes	No	No	120	38	2.12	1.32	300	3.55	840
3	Male	66	25	Yes	Yes	No	No	73	30.2	2.06	1.22	46.3	3.61	613
4	Male	44	29.6	Yes	No	Yes	No	101	36.3	2.21	1.89	64.9	4.19	1216
5	Male	53	27.2	Yes	Yes	No	No	83	25.1	1.81	1.03	152	3.11	688
6	Male	45	27.6	Yes	No	No	No	72	35.5	1.7	2.3	335	4.21	1377
7	Male	40	25.8	Yes	No	Yes	No	87	33.2	1.94	2.15	53.6	3.57	1516
8	Male	33	24.9	Yes	No	No	No	68	27.4	2.27	0.95	54.3	3.29	1748

Abbreviations: BMI, body mass index; DM, Diabetes mellitus; Hb, Hemoglobin; ALB, Albumin; Cr, creatinine; PTH, parathyroid hormone

### Summary of clinical experience based on 8 cases of scrotal leakage

#### Advantages of CT peritoneography in diagnosing these 8 cases

Among the 8 cases, the main clinical presentation was unilateral or bilateral scrotal and penile swelling. Based on physical examination and medical history, dialysate leakage into the scrotum was suspected. Our team promptly performed iopromide CT peritoneography, with an average time to diagnosis ranging from 0.5 to 5 days. The majority of patients underwent examination within 2 days, although in one cases the procedure was delayed due to patient preference (case 7). According to a retrospective review of electronic medical records and telephone follow-up with the patient, it was confirmed that after the initial CT examination, the case 7 independently decided to forgo further diagnostic work-up as the symptoms were deemed tolerable and showed transient improvement.

The results of CT peritoneography in all patients revealed widening of the inguinal canal and high-density fluid extending from the peritoneal cavity into the scrotum. Detailed imaging findings are shown in Fig. 3, with comprehensive radiological descriptions and all images provided in Supplementary File 1. A comparison between conventional non-contrast CT and CT peritoneography was illustrated in Case 8: non-contrast CT on November 2, 2020, showed fluid accumulation in the scrotum; follow-up CT peritoneography on November 3, 2020, confirmed scrotal fluid with increased density and hyperdensity along the left spermatic cord, indicating leakage from the left inguinal region into the scrotum. This demonstrates that iopromide CT peritoneography can not only identify the etiology but also dynamically trace the pathway of contrast leakage, offering clear advantages over conventional non-contrast CT. Tracing the route of edema and leakage aids in guiding further intervention.

Early medical consultation is recommended when scrotal swelling occurs. CT peritoneography should be performed promptly to determine whether dialysate leakage is the cause. Delayed management may lead to serious hernia-related complications. In the management of Case 7, there was a significant delay from the initial report of symptoms on April 4, 2021, to the definitive diagnosis via CT peritoneography on March 13, 2023, which confirmed an inguinal hernia with fluid leakage. A review revealed that during the early symptomatic phase (April 7, 2021), the patient only underwent a plain CT scan, which indicated “widening of the right inguinal canal” (detailed in the attached file). Due to the intermittent nature of the symptoms and the patient’s subjective tolerance, no further contrast imaging was pursued at that time. This case suggests that for scrotal swelling that presents intermittently and is well-tolerated, and when initial imaging shows no emergency indications, a clinical strategy of closely monitoring and prioritizing a trial of nighttime automated peritoneal dialysis (APD) may be considered. This approach is particularly suitable for scenarios judged to involve benign, slowly progressive leakage, as it may help some patients avoid unnecessary invasive diagnostic procedures or surgical interventions. However, if symptoms of scrotal leakage acutely worsen, as seen in the later progression of Case 7, immediate CT peritoneography should be performed upon the onset of unilateral or bilateral scrotal/penile swelling.

The contrast agent employed in this study was iopromide, a non-ionic agent which demonstrates a favorable safety and tolerability profile. Throughout the procedure and during subsequent follow-up, all patients were closely monitored for clinical manifestations, including abdominal pain, fever, allergic symptoms, signs of peritonitis, as well as changes in the color and characteristics of the peritoneal dialysate. Concurrently, safety was assessed via laboratory parameters, including relevant hematological tests and routine analysis of ascitic fluid. Among the eight patients included, no discernible adverse reactions attributable to the contrast agent were observed. The specific monitoring indicators and results are detailed in Supplementary File 2 (Follow-up Safety Monitoring Record).

#### Features of patients with scrotal leakage

The baseline characteristics of the eight patients included in this study are summarized in Table 1. The mean BMI of this cohort was 25.64 ± 2.47 kg/m². All patients had concomitant hypertension. The mean serum albumin level was 32.93 ± 4.87 g/L. Laboratory tests also indicated the presence of anemia (mean hemoglobin: 89.63 ± 19.65 g/L), electrolyte disturbances (mean serum calcium: 2.02 ± 0.19 mmol/L, serum phosphorus: 1.53 ± 0.52 mmol/L, serum potassium: 3.57 ± 0.44 mmol/L), and secondary hyperparathyroidism (mean iPTH: 165.26 ± 130.31 pg/mL). Renal function indices at admission revealed a mean creatinine level of 1203.00 ± 439.07 µmol/L. Some patients had a history of abdominal surgery or diabetes.

It should be noted that, given the limited sample size (*n* = 8) of this study, the data presented here are primarily descriptive. Previous literature suggests that factors such as age, history of abdominal surgery, nutritional status, electrolyte levels, and diabetes may be associated with the occurrence of peritoneal dialysis-related complications [13, 14]. The features observed in this case series provide a reference for further research into PD mechanical complications, particularly scrotal leakage. However, the exact associations between these characteristics and scrotal leakage require validation in larger-scale studies.

#### Clinical management timeline of patients with dialysate scrotal leakage

We retrospectively analyzed the full management course of the 8 patients with scrotal swelling. Following the onset of symptoms, the peritoneal dialysis regimen was promptly adjusted: all patients were switched to daytime dry abdomen and nighttime automated peritoneal dialysis (APD), with a specific prescription of 10–15 L of dialysate over 8–10 h. Iopromide CT peritoneography was performed for diagnostic confirmation. After diagnosis of scrotal leakage, surgical consultation was obtained. Based on surgical indications, 7 patients underwent laparoscopic transabdominal preperitoneal (TAPP) repair, and 1 patient underwent totally extraperitoneal (TEP) repair. Within the first month post-surgery, transitional therapy involving APD or temporary hemodialysis was implemented. After one month, all 8 patients resumed CAPD. During long-term follow-up, 5 patients remained on CAPD, 2 received combined CAPD and hemodialysis, and 1 underwent kidney transplantation and ceased dialysis. None of the patients experienced recurrence of scrotal leakage. Moreover, no adverse reactions related to peritoneography were observed (details were shown in Supplementary File 2, Follow-up Safety Monitoring Record). A detailed clinical management timeline for these cases is provided in Fig. 4; Table 2.

**Table 2:** Clinical management timeline of peritoneal dialysis-related scrotal leak cases

Case Number	Peritoneal Dialysis Catheter Insertion Time	Hospital Admission Time	Time from Catheter Insertion to Onset (Days)	Time of Symptom Onset	Time Taken for Diagnosis (Days)	Diagnosis Time		Surgery Time	Time from Diagnosis to Surgery (Days)	Surgical Procedure	Postoperative Treatment
1	2021-6-1	2025-7-28	1518	1 week before admission: Nausea, dizziness, fatigue, left scrotal swelling	2.0	2025-7-30		2025-8-2	3.0	Laparoscopic bilateral inguinal indirect hernia repair with preperitoneal mesh (TAPP)	APD
2	2023-2-22	2024-1-8	318	2024-01-06: Bilateral scrotal enlargement after lifting heavy objects	1.0	2024-1-9	2024-1-15		6.0	(Bilateral) Laparoscopic Transabdominal Preperitoneal Patch Plasty for inguinal hernia (TAPP)	APD
3	2025-2-13	2025-4-2	23	Over 3 weeks before admission: Bilateral scrotal enlargement	1.0	2025-4-3	2025-4-10		7.0	Laparoscopic Totally Extraperitoneal Patch Plasty for inguinal hernia (TEP), Bilateral	APD
4	2024-12-11	2025-5-7	147	1 day before admission: Scrotal edema with pain	0.5	2025-5-7	2025-5-9		2.0	Laparoscopic Transabdominal Preperitoneal Patch Plasty for inguinal hernia (TAPP)	APD
5	2024-6-26	2024-8-16	49	10 days before admission: Bilateral lower limb edema, 3 days before admission: Scrotal edema	3.0	2024-8-19	2024-9-3		14.0	Laparoscopic Transabdominal Preperitoneal Patch Plasty for inguinal hernia (TAPP) (Bilateral indirect inguinal hernia) + Laparoscopic adhesiolysis	APD
6	2022-7-15	2024-2-23	589	Recent history of recurrent scrotal swelling, self-reducible, impulse felt in right groin when coughing	2.0	2024-2-25	2024-3-1		4.0	Laparoscopic Transabdominal Preperitoneal Patch Plasty for inguinal hernia (TAPP) (Right indirect and direct hernia)	low-dose APD (poor dialysis efficacy), switched to temporary hemodialysis irrigation
7	2020-9-29	2023-3-9	188	2021-4-4: Scrotal enlargement	4.0	2023-3-13	2023-3-24		11.0	Laparoscopic Transabdominal Preperitoneal Patch Plasty for inguinal hernia (TAPP) (Right indirect hernia)	Temporary hemodialysis
8	2017-3-6	2020-10-29	1303	1 month before admission: Scrotal enlargement	5.0	2020-11-3	2020-11-9		6.0	Anterior approach left inguinal hernia extraperitoneal repair	Temporary hemodialysis

Abbreviations: APD, automated peritoneal dialysis

## Discussion

Peritoneal dialysis, as one of the renal replacement therapies for end-stage renal disease, has been widely adopted worldwide since it was first introduced by Popovich et al. in 1976 [1, 2]. Dialysate leakage, often resulting from increased intra-abdominal pressure during peritoneal dialysis, may arise from congenital or acquired defects [14–16]. Based on the timing of occurrence, leakage can be classified as early or delayed. Early leakage occurs within 30 days after catheter placement, is usually related to the catheter insertion procedure, presents as external leakage at the puncture site, and is readily detectable and manageable. Delayed leakage occurs more than 30 days after catheter placement and is most commonly associated with mechanical injury or surgical trauma to the peritoneum, manifesting as internal leakage (e.g., into the pleural cavity, abdominal wall, or external genitalia). The scrotal leakage cases included in this study represent a common form of internal genital leakage. Among peritoneal dialysis-related complications, umbilical hernia is most frequently reported, followed by inguinal, incisional, and epigastric hernias [16]. Dialysate-induced hydrocele often represents an early stage of inguinal hernia [17], which is clinically subtle and easily overlooked. Conventional physical examination and ultrasound often fail to detect it, underscoring the importance of auxiliary imaging for early and accurate diagnosis.

Therefore, we conducted a retrospective analysis of 8 patients with confirmed dialysate scrotal leakage diagnosed by CT peritoneography between August 2020 and August 2025. We aimed to evaluate the feasibility and diagnostic reliability of CT peritoneography for early confirmation, particularly its ability to identify the site of peritoneal defect and trace the pathway of dialysate leakage. We collected clinical baseline characteristics, peritoneal dialysis initiation time, time of scrotal leakage onset, and the full diagnostic and treatment timeline. Furthermore, we explored common features for scrotal leakage in PD patients, summarized our diagnostic and therapeutic approach in a timeline format, and shared clinical experience to aid in the management of this condition.

### Value of CT peritoneography in diagnosing scrotal leakage in peritoneal dialysis

Using iopromide CT peritoneography, we confirmed the diagnosis of dialysate scrotal leakage in all 8 patients presenting with scrotal or penile swelling, demonstrating the value of this technique in identifying leakage pathways and determining etiology. Compared with conventional non-contrast CT, iopromide-enhanced CT peritoneography dynamically visualized the migration of contrast medium from the peritoneal cavity through the inguinal canal into the scrotum, clearly revealing the anatomic route of leakage and providing precise guidance for clinical intervention. This advantage was particularly evident in Case 8: while non-contrast CT only indicated scrotal fluid accumulation, contrast CT further identified the leakage origin and hyperdensity along the spermatic cord, highlighting its superiority in high-resolution dynamic tracing. Previous studies have also shown that CT peritoneography clearly depicts the entry of contrast-enhanced dialysate into the inguinal canal or scrotum in cases complicated with inguinal hernia [18]. Similarly, in incisional or umbilical hernias, it can clearly demonstrate the presence of contrast-containing dialysate within the hernial sac and delineate the size and location of the hernia orifice.

Moreover, our results emphasize the necessity of early examination. For instance, in Case 6, symptoms presented 589 days after catheter insertion, leading to a prompt diagnosis of an inguinal hernia and hydrocele within 2 days. This case suggests that a prolonged period without intervention, even if asymptomatic, may ultimately result in complex hernia and related complications. Early tracking of contrast leakage pathways with CT peritoneography allows clearer localization of the defect, facilitating prompt diagnosis and surgical treatment, thereby preventing progression to content-containing hernia. Regarding safety, iopromide, as a non-ionic contrast agent, demonstrated good safety and tolerability in this patient group, with no observed nephrotoxicity or other adverse reactions. This finding is consistent with previous reports on its use in populations with renal impairment [18, 19]. In comparison with other diagnostic modalities, we also conducted a comparative analysis based on previously published studies, with detailed information presented in Fig. 2. Therefore, we recommend early performance of iopromide CT peritoneography when dialysate leakage is clinically suspected. CT peritoneography demonstrated its capability to dynamically trace the pathway of contrast medium from the peritoneal cavity through the inguinal canal into the scrotum, thereby providing precise anatomical localization of leakage sites. This technique offers clear advantages over conventional non-contrast CT in terms of dynamic visualization and high-resolution tracing of leakage routes. Early examination is recommended upon clinical suspicion of dialysate leakage to facilitate prompt diagnosis and guide subsequent intervention.

### Clinical considerations regarding delayed dialysate leakage-related scrotal leak

Sites of delayed dialysate leakage include the abdominal wall, external genitalia, hernial orifices, pleural cavity, and retroperitoneal space [20], often occurring at areas of abdominal wall weakness. Reported high-risk factors for delayed leakage in the literature include a history of multiple abdominal surgeries, multiparity, obesity, long-term corticosteroid use, and repeated abdominal operations [15, 21, 22]. The combination of these factors with increased intra-abdominal pressure during dialysis can contribute to dialysate leakage.

In the case series included in this study, certain characteristics were observed, such as overweight (mean BMI 25.64 ± 2.47 kg/m²), hypertension (present in all patients), and hypoalbuminemia (mean 32.93 ± 4.87 g/L). It is noteworthy that previous literature has indicated that factors such as age, history of abdominal surgery, nutritional status (e.g., albumin levels), and electrolyte disturbances may be associated with the occurrence of peritoneal dialysis-related complications [13, 14]. The features observed in this case series are consistent with some of the potential risk factors for PD mechanical complications described in the literature. However, it must be emphasized that, given the limited sample size (*n* = 8) of this study, the data here are presented in a descriptive manner to provide a clinical profile of this specific patient group and should not be interpreted as establishing causal relationships between these characteristics and scrotal leakage.

Furthermore, sudden elevations in intra-abdominal pressure, triggered by activities such as coughing, straining, jumping, or heavy lifting, can increase pressure levels up to 300 cm H₂O [23], which are known triggers of dialysate leakage. Therefore, in clinical management, enhancing patient education on avoiding or minimizing such activities that increase intra-abdominal pressure is crucial for preventing leakage. Based on existing evidence in the literature, screening patients with known risk factors (e.g., obesity, history of abdominal surgery) and implementing preventive strategies, including intra-abdominal pressure management, may help reduce the risk of developing scrotal leakage and other peritoneal dialysis-related leakage events. Common clinical features observed in this case series, such as overweight, uncontrolled hypertension, and hypoalbuminemia, align with factors previously associated with peritoneal integrity compromise. These findings highlight the importance of monitoring these parameters in clinical practice. Additionally, patients should be advised to avoid activities that cause sudden increases in intra-abdominal pressure.

### Insights into the diagnosis and management of peritoneal dialysis-related scrotal leakage

In this study, we implemented a systematic multidisciplinary strategy for managing patients with scrotal swelling, emphasizing rapid diagnosis and individualized treatment, which was shown in Fig. 4. Upon symptom recognition, all patients immediately transitioned to a regimen of daytime dry abdomen and nighttime automated peritoneal dialysis (APD). This approach aimed to reduce intra-abdominal pressure and dialysate accumulation, thereby mitigating leakage and alleviating symptoms. If response remained suboptimal, temporary hemodialysis was introduced to facilitate subsequent interventions.

After confirming scrotal leakage, surgical consultation was promptly obtained. Laparoscopic hernia repair (either TAPP or TEP) was performed based on individual anatomical characteristics and clinical conditions. These minimally invasive techniques significantly reduced trauma and recurrence risk while supporting subsequent recovery and dialysis continuity. During the first postoperative month, patients were maintained on APD or temporary hemodialysis to avoid early increases in intra-abdominal pressure that could compromise repair integrity. After one month, all patients successfully resumed CAPD, confirming the safety and feasibility of this transitional protocol.

Long-term follow-up revealed no recurrence in any patient. Dialysis modalities were flexibly adjusted according to individual clinical needs (e.g., CAPD, combined CAPD-hemodialysis, or kidney transplantation). A detailed diagnostic-therapeutic timeline is provided in Fig. 4, summarizing the clinical course and management adaptations for the described cases.

The implemented management strategy involved immediate transition to automated peritoneal dialysis with daytime dry abdomen upon symptom recognition, followed by confirmatory CT peritoneography. Surgical repair was subsequently performed using minimally invasive techniques, with all patients successfully resuming continuous ambulatory peritoneal dialysis after a one-month transition period. This approach was associated with resolved leakage and no recurrence during follow-up.

### Strengths and limitations of this study

The strength of our study lies in being the first to systematically summarize and validate the integrated application value of a CT peritoneography-centered multidisciplinary approach in the management of peritoneal dialysis-associated scrotal leakage. We observed specific clinical characteristics within our case series, including overweight, uncontrolled hypertension, and hypoalbuminemia, and implemented a holistic “imaging-to-intervention” strategy. This integrated approach enabled precise visualization of leakage pathways, providing a reliable basis for early clinical intervention. Furthermore, all patients underwent standardized laparoscopic repair and individualized dialysis regimen adjustment. During long-term follow-up, no recurrence was observed in the case series, and outcomes were favorable overall. These clinical observations are consistent with the potential application of this management pathway based on our experience.

However, several limitations should be acknowledged. First, as a single-center retrospective study with a small sample size (*n* = 8), the findings may be subject to selection bias. Second, although we conducted a systematic review and comparative summary of other diagnostic modalities, the lack of direct comparisons with alternative diagnostic methods (e.g., ultrasound or radionuclide imaging) or surgical techniques makes it difficult to fully evaluate the advantages and disadvantages of different strategies. Additionally, although the follow-up period indicated long-term effectiveness, larger sample sizes and longer observation periods are needed to confirm recurrence rates and treatment durability, future studies need to validate diagnostic accuracy through more rigorous research designs. Future prospective multicenter studies are warranted to further refine the diagnostic workflow and surgical strategies and to explore risk stratification-based preventive management pathways.

## Conclusion

This case series demonstrates that CT peritoneography provides precise anatomical delineation of dialysate leakage pathways, thereby enabling targeted interventions. The described multidisciplinary management approach, which incorporates imaging confirmation, dialysis regimen adjustment, and laparoscopic repair, was associated with successful resolution of scrotal leakage complications. Furthermore, the frequently observed clinical features of overweight, hypertension, and hypoalbuminemia in this patient population warrant particular attention in clinical management.

## Supplementary Information

Below is the link to the electronic supplementary material.

Supplementary Material 1

Supplementary Material 2

## Data Availability

The data described in this article can be freely and openly accessed in this article supplement material.

## Abbreviations

BMI: Body mass index
SBP: Systolic blood pressure
DBP: Diastolic blood pressure
Cr: Creatinine
PTH: Parathyroid hormone
APD: Automated peritoneal dialysis
