# Supplementary material for: Iopromide CT peritoneography for diagnosis and management of dialysate scrotal leakage in continuous ambulatory peritoneal dialysis
Source: BMC Nephrol. 2026 Mar 27;27:288. doi: 10.1186/s12882-026-04901-5 (PMC13147658; doi:10.1186/s12882-026-04901-5)
Supplement: Supplementary file 1 — Supplementary Material 1 [file 12882_2026_4901_MOESM1_ESM.docx]

Supplementary Table. Follow-up Safety Monitoring Record

| Case | Abdominal Pain | Allergy | Ascitic Fluid Routine Leukocytes (10*6/L) | Peritoneal Dialysate Color | Leukocytes  (10*9/L) | Blood Neutrophils (10*9/L) |
| --- | --- | --- | --- | --- | --- | --- |
| 1 | No | No | 35 | clear | 5.03 | 3.69 |
| 2 | No | No | 5 | clear | 6.63 | 3.76 |
| 3 | No | No | 13 | clear | 9.17 | 5.9 |
| 4 | No | No | 39 | clear | 6.02 | 3.83 |
| 5 | No | No | 3 | clear | 5.96 | 4.83 |
| 6 | No | No | 10 | clear | 4.5 | 2.51 |
| 7 | No | No | 5 | clear | 6.42 | 4.22 |
| 8 | No | No | 10 | clear | 6.38 | 4.03 |
