# Supplementary material for: Iopromide CT peritoneography for diagnosis and management of dialysate scrotal leakage in continuous ambulatory peritoneal dialysis
Source: BMC Nephrol. 2026 Mar 27;27:288. doi: 10.1186/s12882-026-04901-5 (PMC13147658; doi:10.1186/s12882-026-04901-5)

Supplement Appendix 1

**Iopromide CT Peritoneography for Diagnosis and Management of Dialysate Scrotal Leakage in Continuous Ambulatory Peritoneal Dialysis: A Report of 8 Cases**

Zhibing Xie^1#^, Huyan Yu^1#^, Jian Lin^1*^, Qing Ye^1*^

1. Department of Nephrology, Zhongshan City People's Hospital, Zhongshan 528403, China.

# Contribute equally to this work as first author

* Corresponding author:

Qing Ye, Email: yeqing2318@aliyun.com

**Imaging Data of CT Peritoneography in Peritoneal Dialysis Patients with Scrotal Leak**

**Case 1**

Bilateral inguinal canals are widened, with contrast-enhanced fluid density extending from the peritoneal cavity into the scrotum.


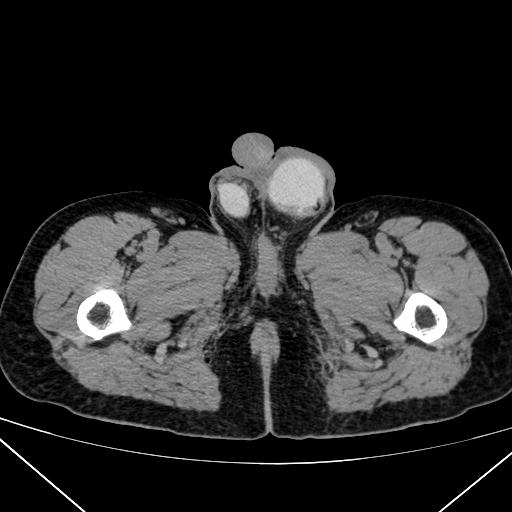

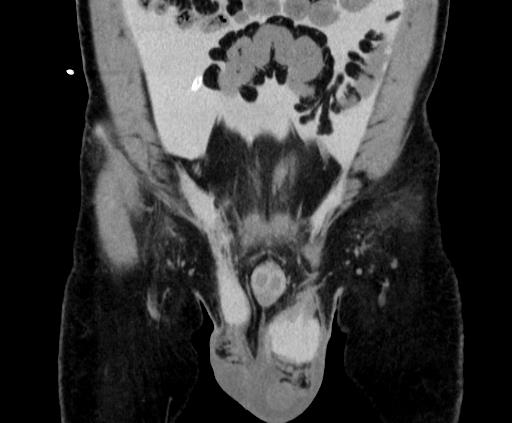


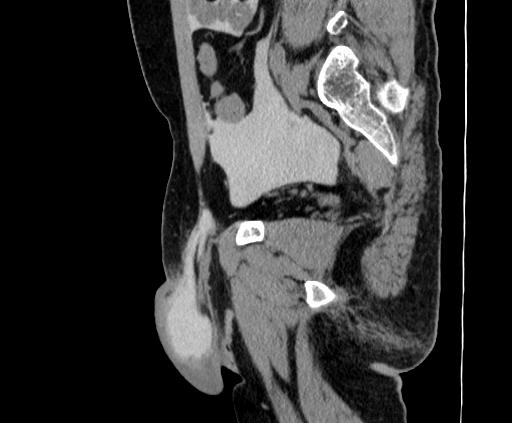


**Case 2**

The right inguinal orifice is widened, with a small amount of fluid tracking along the dilated right inguinal canal into the tunica vaginalis of the right scrotum.
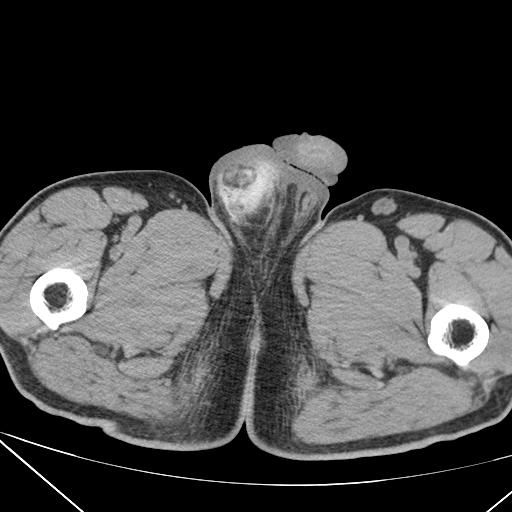

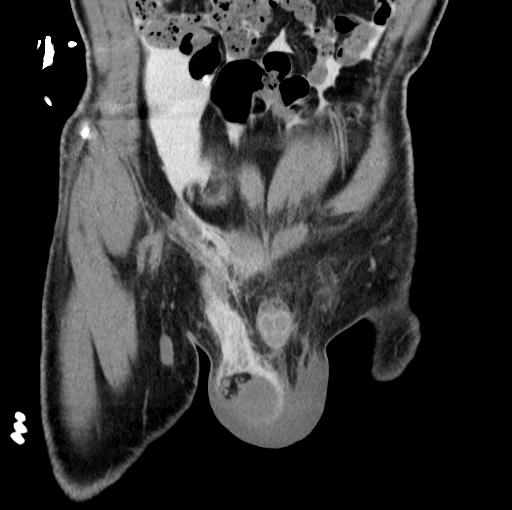


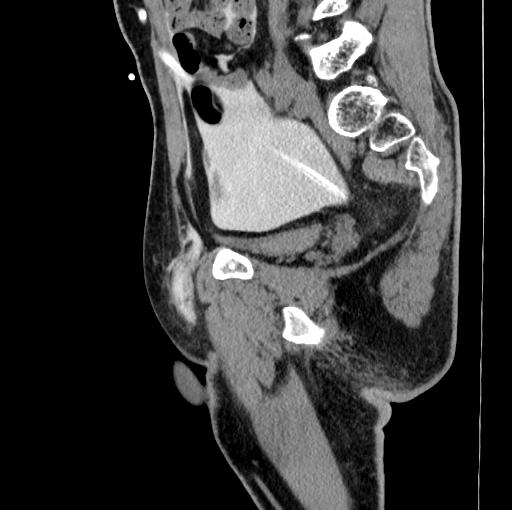


**Case 3**

The left inguinal canal is widened with contrast medium extending into the scrotum. Bilateral scrotal enlargement is noted, accompanied by patchy high-density opacities within the scrotal cavity.


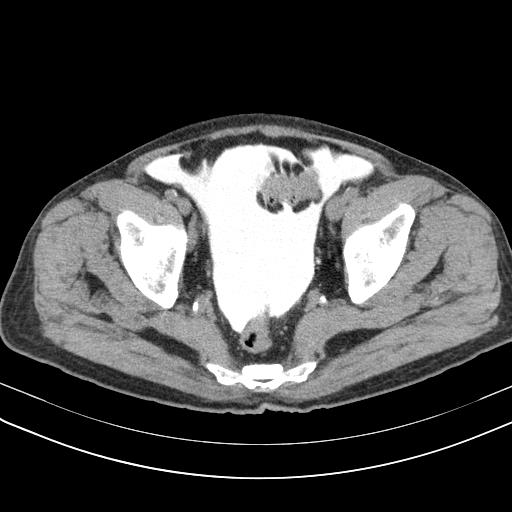


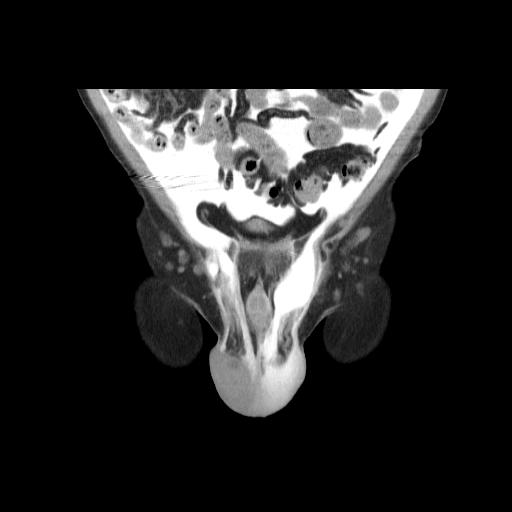

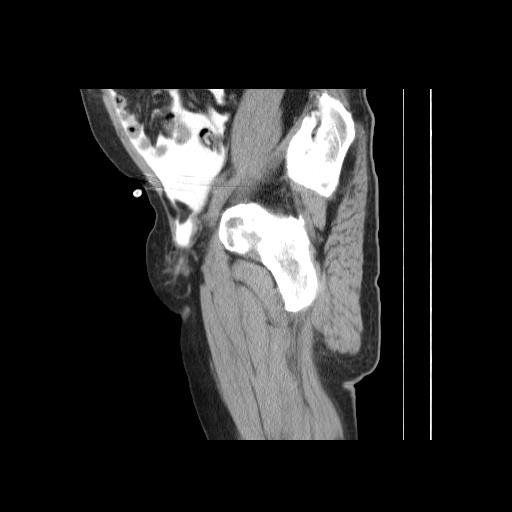


**Case 4**

The left inguinal canal is dilated, with contrast medium accumulation observed within the left scrotum. Soft tissue edema is noted in the perineal region.


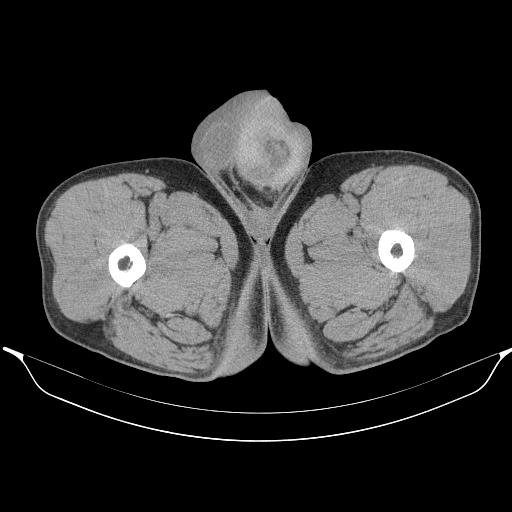

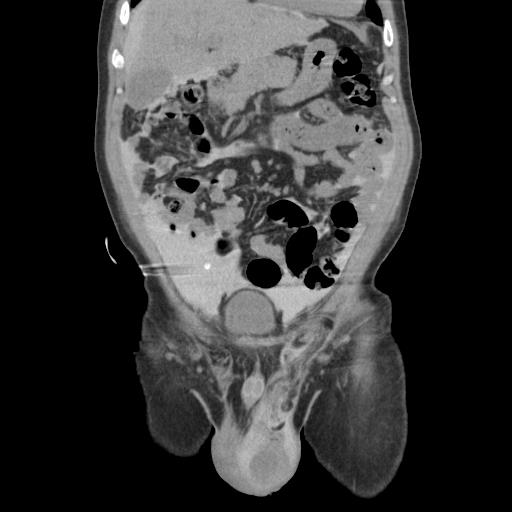


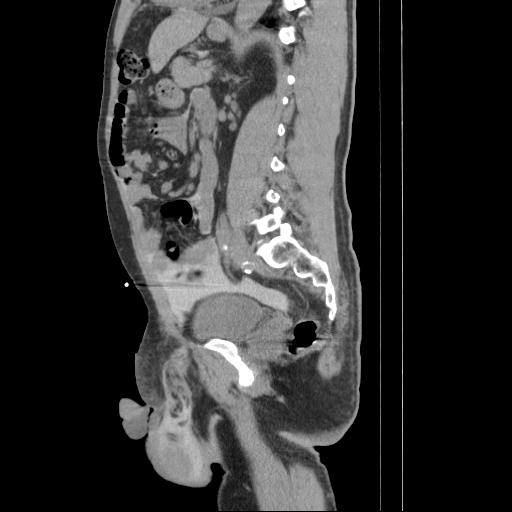


**Case 5**

The left inguinal canal is widened, with peritoneal fluid tracking along it into the scrotum.


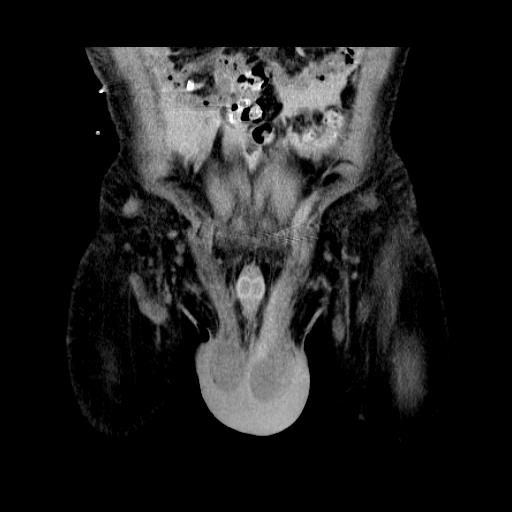


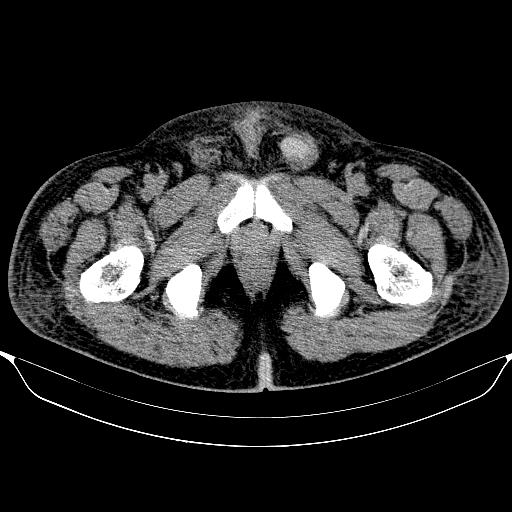


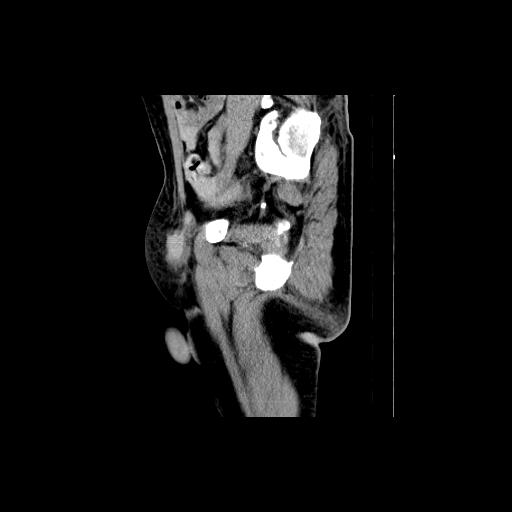


**Case 6**

Right inguinal canal hernia is observed, containing fat tissue. A small amount of fluid is noted around the hernia. Bilateral testicular hydroceles are present.


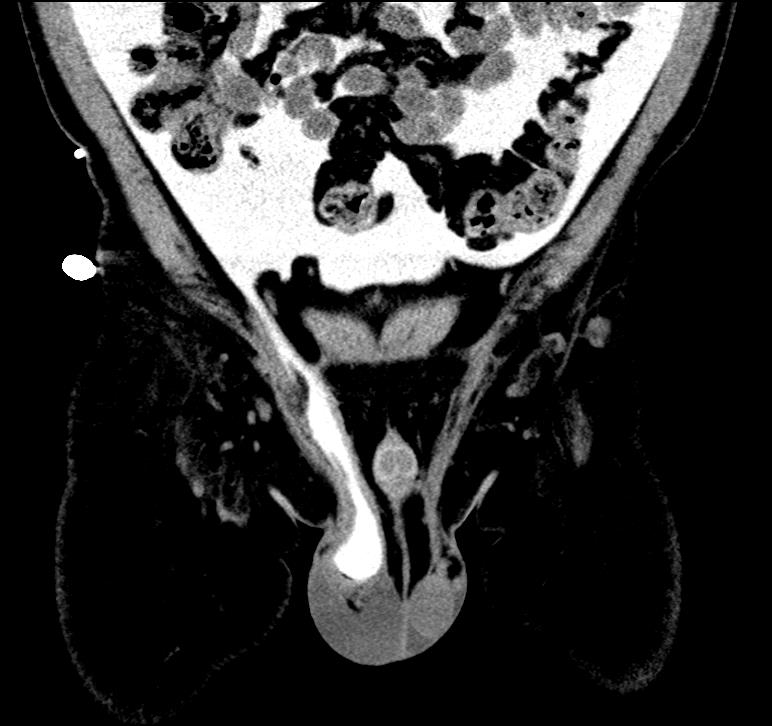

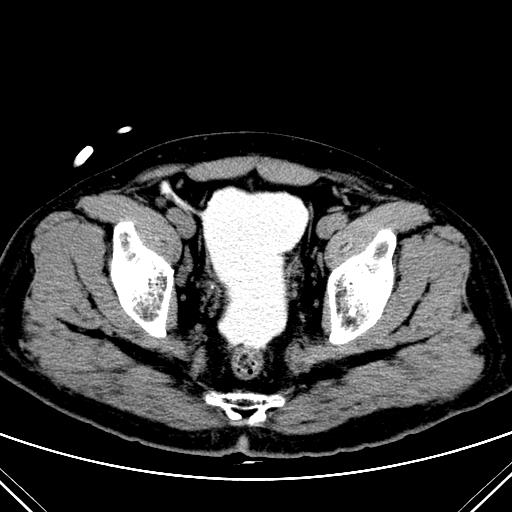


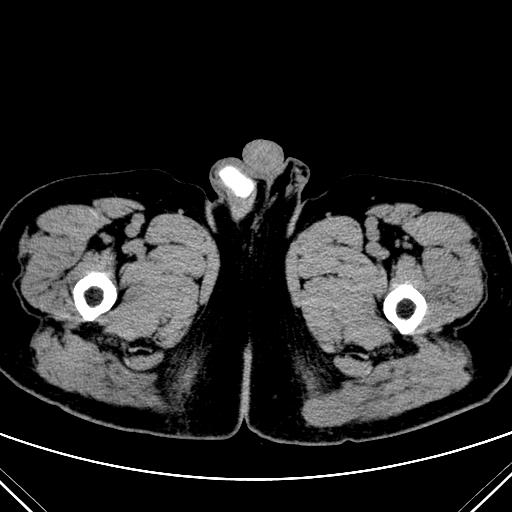

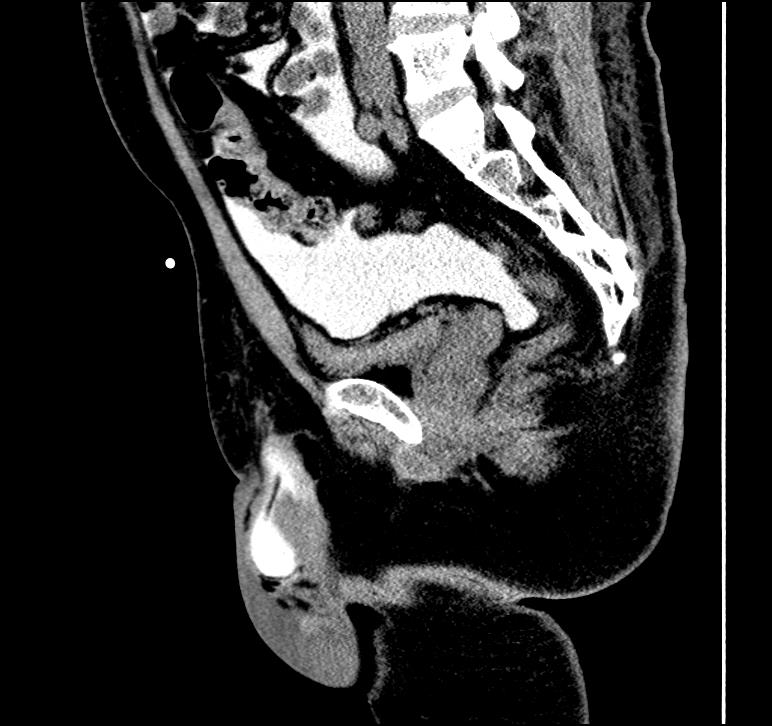


**Case 7**

A non-contrast CT scan was performed on April 7, 2021, with the following finding: "widening of the right inguinal canal."


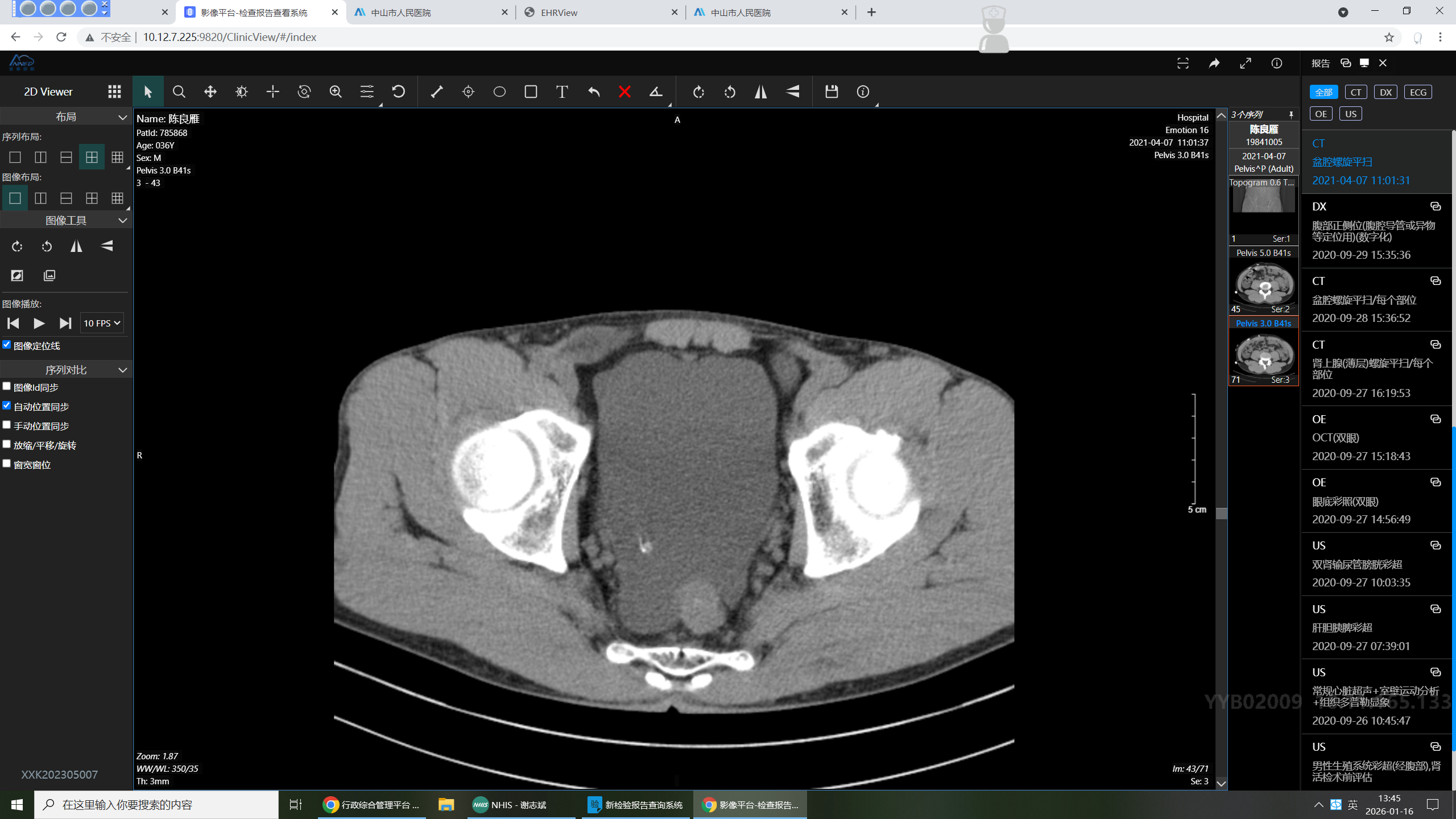


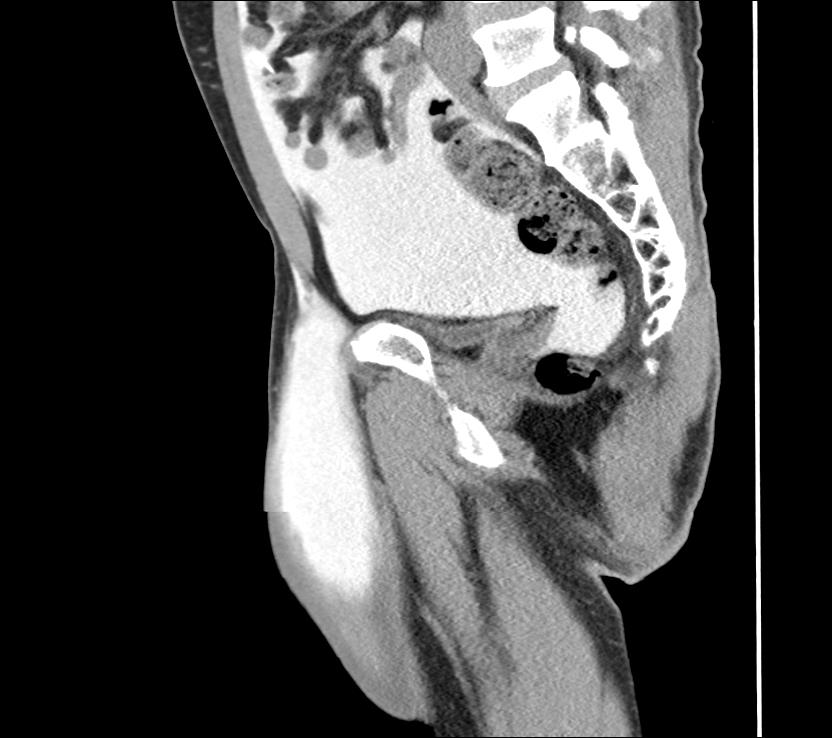

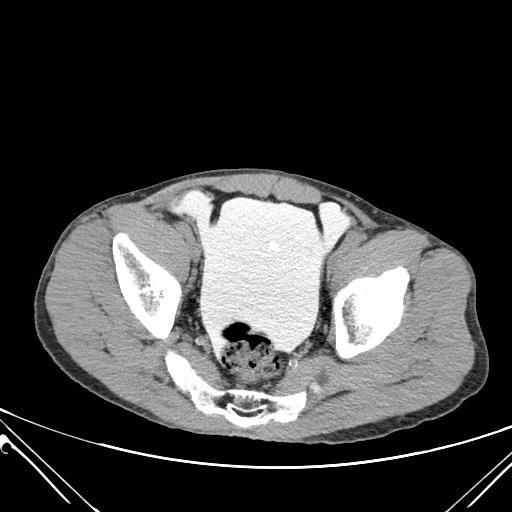

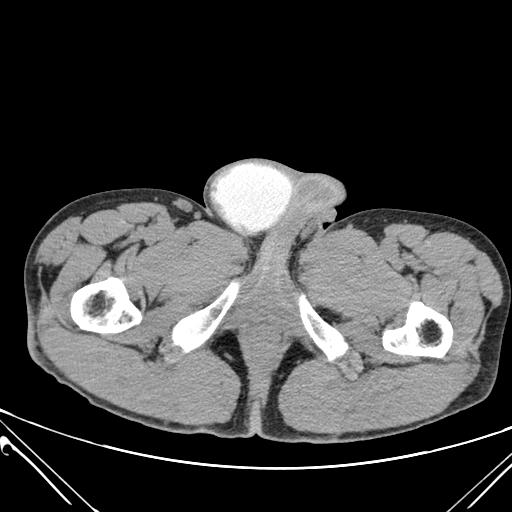
A right inguinal hernia is identified, with a hernia sac measuring 75mm x 59mm, extending downward into the scrotum. No bowel loops are herniated. A right testicular hydrocele is present.


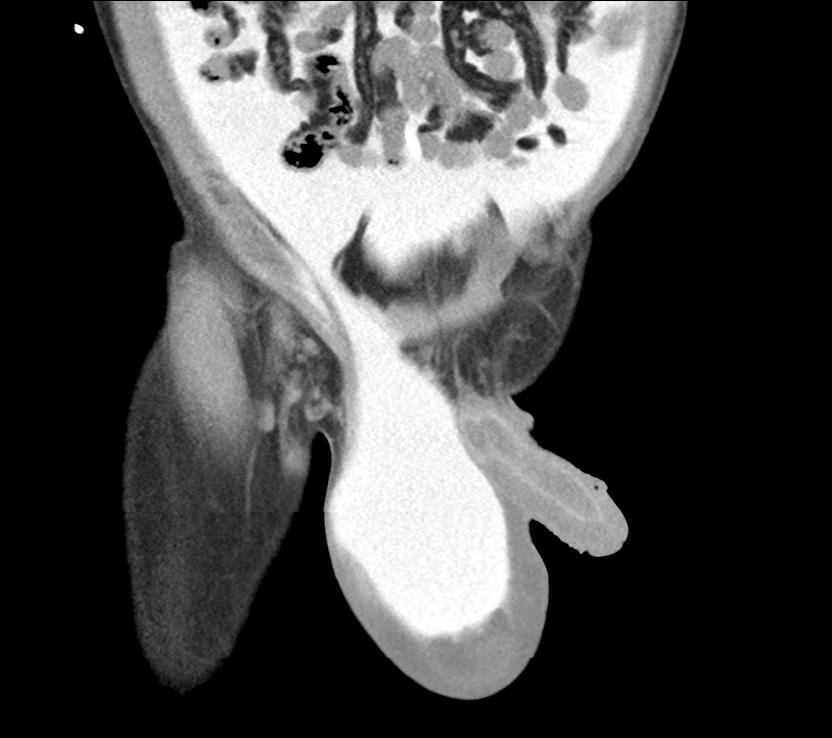


**Case 8**


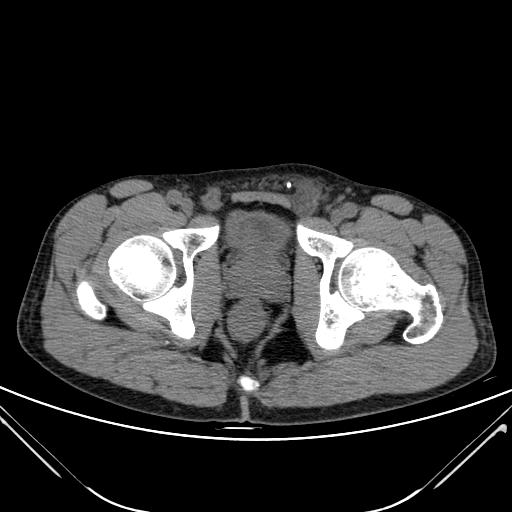

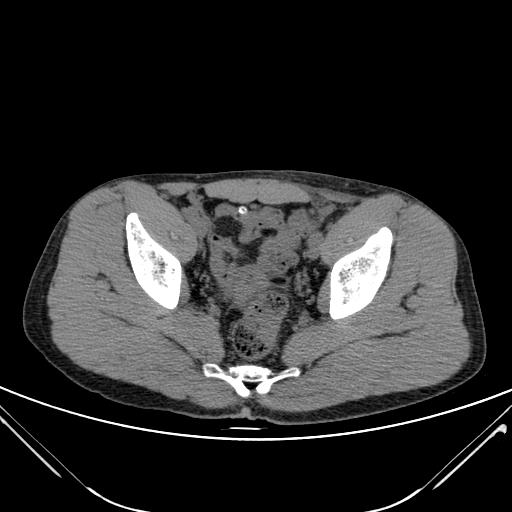
Non-contrast CT scan (2020-11-02): Fluid collection is noted within the scrotum.


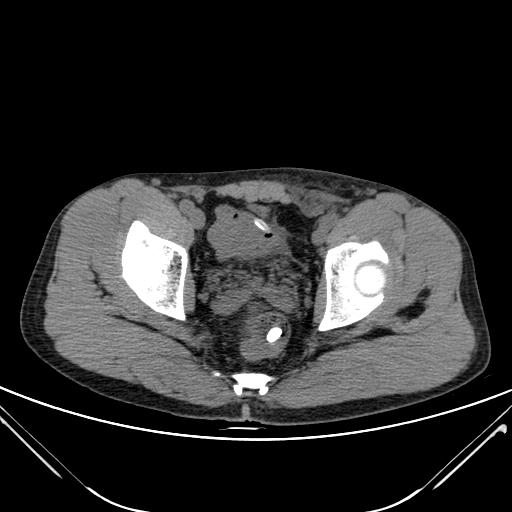


**CT peritoneography with intraperitoneal contrast (2020-11-03) demonstrates:**

1.Scrotal fluid collection with increased density, consistent with contrast accumulation within the scrotum.

2.Increased density along the course of the left spermatic cord, indicating contrast tracking from the left inguinal region into the scrotum.


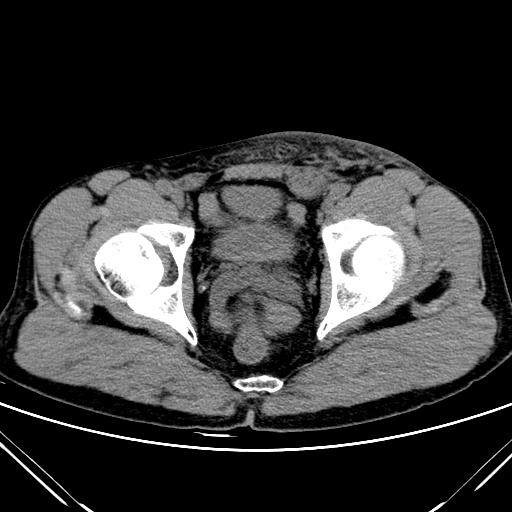
3.Subcutaneous edema along the anterior pelvic wall.


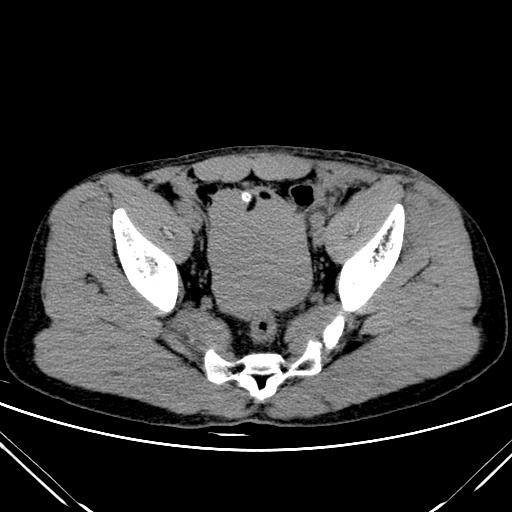

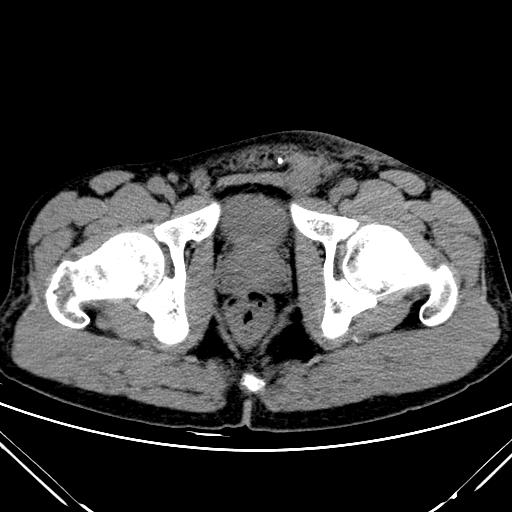

Supplement: Supplementary file 2 — Supplementary Material 2 [file 12882_2026_4901_MOESM2_ESM.docx]
